# Determinants of Tuberculosis Infection among Adult HIV Positives Attending Clinical Care in Western Ethiopia: A Case-Control Study

**DOI:** 10.1155/2013/279876

**Published:** 2013-10-08

**Authors:** Hatoluf Melkamu, Berhanu Seyoum, Yadeta Dessie

**Affiliations:** ^1^Department of Public Health, Colleges of Health and Medical Sciences, Haramaya University, P.O. Box 235, Harar, Ethiopia; ^2^Department of Medical Laboratory Science, Colleges of Health and Medical Sciences, Haramaya University, Harar, Ethiopia

## Abstract

There has been a drastic rise of tuberculosis (TB) infection across the world associated with the pandemic occurrence of HIV/AIDS. There are various determinants factors that increase the chance of TB infection among HIV positives (TB/HIV confection) that varies contextually. This study aimed to assess the determinants of TB/HIV coinfection among adult HIV positives attending clinical care at two public health facilities in Nekemte, western Ethiopia. Unmatched case-control study was conducted from December 26, 2011, to February 29, 2012. Cases were 123 TB infected HIV positives, and controls were 246 non-TB infected HIV positives. Being divorced/widowed AOR = 3.02, 95% CI (1.70, 7.88), not attending formal education AOR = 4.32, 95% CI (2.20, 14.15), being underweight (BMI < 18.5 kg/m^2^) AOR = 3.87, 95% CI (2.18, 6.87), having history of diabetic mellitus AOR = 3.63, 95% CI (1.33, 9.94), and being in advanced WHO HIV/AIDS clinical staging AOR = 2.29, 95% CI (1.32, 3.98), were determinant factors associated with TB/HIV co-infection. Having a separate kitchen AOR = 0.48, 95% CI (0.28, 0.81) showed protective role. For most of these determinants interventions can be made at individual and institutional levels, whereas, factors like education and nutrition need societal level integrations.

## 1. Introduction 

Tuberculosis (TB) is one of the world's most common cause of death in the era of human immunodeficiency virus (HIV). It is among the leading causes of death for people living with HIV (PLWHIV) which shares about twenty-five percent of all causes of the deaths [1]. TB and HIV are called a “deadly duo” as HIV weakens the immune system and makes them more susceptible for TB infection. On the other hand, TB increases the progression of HIV to AIDS stage [2].

Globally, more than 13 million people are TB/HIV coinfected. Of these, about seventy percent are living in sub-Saharan Africa [3]. Ethiopia ranked seventh among the twenty-two high TB burden countries in the world [4]. Hospital data indicated that TB is the leading cause of morbidity and the third cause of hospital admissions in the country [5].

Studies indicated that certain HIV-infected people develop TB, while others do not. This phenomenon iterates that being HIV positive is not a mere factor for being infected with TB, and there are various determinants factors that contribute to the TB/HIV co-infection [6–8]. These factors vary contextually which necessitate conducting the present study. Therefore, the aim of this study was to identify the determinants of TB/HIV co-infection among HIV-positive adults attending clinical care at two public health facilities in Nekemte, western Ethiopia.

## 2. Methods

### 2.1. Study Setting

This study was conducted in one public hospital and a health center found in Nekemte which is the capital town of east Wollega Zone of Oromia Regional State. The town is located at about 331 km from Addis Ababa (the capital city) to the west of country. The two public health facilities are providing free ART services in the town since 2005 and 2006, respectively. In 2010/11, a number of clients attending HIV clinical care were 8,760 PLHIV in the hospital and 1,660 in the health center. Overall, 184 TB/HIV co-infected patients were in clinical care during the commencement of this study (146 in the hospital and 38 in the health center) [9]. This study was conducted between December 26, 2011, and February 29, 2012.

### 2.2. Participants

The design for this study was unmatched case control. Cases were TB/HIV co-infected patients whereas controls were non TB infected HIV-positive patients. The inclusion criteria for the study were being 15 years and above, TB/HIV coinfected (PTB, EPTB, mixed or disseminated) for cases and PLHIV with non-TB infected were selected for controls. Those patients who were seriously ill, had a mental problem and unable to give consent, transferred to other facilities outside of the study area, and not confirmed as TB were excluded from the study.

To select the study subjects, lists of cases and controls were prepared using unique identification numbers from records found in ART clinics by computer-generated random numbers. Cases and controls were selected proportional to a number of patients on clinical care in the health facilities during the study period. With the procedure, 123 TB co-infected and 246 non-TB infected HIV positives were selected for cases and controls, respectively.

### 2.3. Measurements

Being tuberculosis co-infected or not co-infected is the dependent variable for the study. The independent variables include sociodemographic and economic characteristics (age, sex, income, educational status, and marital status), host and clinical related characteristics (WHO clinical stage, CD4 count, past history of TB, body mass index (BMI), smoking, history of asthma, and diabetic mellitus), and environmental related characteristics (overcrowding, wall and floor type of residential house).

### 2.4. Data Collection

Five trained nurse counsellors (3 for the hospital and 2 for health centres) who were fluent speakers of the local languages (Afan Oromo and Amharic) collected the data. Pretested structured questionnaire was used to collect the data. The data collection was through face-to-face interview approach. The interview was made in a separate room to ensure privacy and to facilitate discussions between the interviewer and the respondent. Other clinical characteristics data were retrieved from each patient record. The overall data collection process was supervised by two supervisors (public health professionals) and the investigators.

### 2.5. Data Analysis

Data were cleaned and entered to Epi info version 3.5.3 software. Analysis was made using SPSS version 16.0 software package. Descriptive statistics were used to assess normality, outliers and identify missing values. Bivariate analysis was performed to see the association between the dependent and independent variables. To measure the strength of association, odds ratio with a 95% confidence level was calculated. Multivariable logistic regression was done by entering all variables with *P* value less than 0.25 in the bivariate analysis. Finally, unconditional logistic regression with backward method was used to control possible confounders and to identify the determinant factors associated with TB/HIV co-infection.

### 2.6. Ethical Considerations

The study was reviewed and approved by the Institutional Research Ethics Review Committee of the Colleges of Health and Medical Sciences, Haramaya University. Moreover, written consent was obtained from all study participants before commencement of the study. 

## 3. Results

### 3.1. Sociodemographic Characteristics

A total of 357 (119 cases and 238 controls) were included in the study. The response rate was 96.7%. More than half 192 (53.8%) of them were females. The median (IQR) age was 35 years (IQR = 10). Three hundred twenty-seven (91.6%) were urban dwellers and 251 (70.3%) attended formal education. In bivariate analysis, those widowed/divorced were 2.7 times more likely to develop TB than those who were single or married, OR = 2.7, 95% CI (1.49, 5.34). Those who had not attended formal education were 4.5 times more likely to develop TB than those who attended tertiary level education, OR = 4.55, 95% CI (2.09, 9.90) (Table 1).

### 3.2. Host and Clinical Related Characteristics

Sixteen of the cases (13.4%) and 10 (4.2%) of the controls had been experienced diabetic mellitus. Those with history of diabetic mellitus were more likely infected with TB, OR = 3.53, 95% CI (1.55, 8.07) though did not reach significance after controlling for the confounders. Low CD4 cell count (<200 cell/mm^3^) was significantly associated with TB infection, OR = 2.35, 95% CI (1.23, 4.48). Compared to the respondent who had CD4 count greater than 500 cells/mm^3^, those who had CD4 count below 200 and 200–500 cell/mm^3^ were 2.3 and 1.4 times more likely to develop TB infection, respectively. Those who were in WHO clinical stages three and four were two times more likely to develop TB than those in stage one and two OR = 2.09, 95% CI (1.33, 3.28). Those who had hemoglobin level of less than 10 mg/dL, OR = 2.96, 95% CI (1.28, 6.80), were more likely to develop tuberculosis than those who had 12.5 mg/dL and above hemoglobin level. Having BMI less than 18.5 m^2^/kg was associated with TB infection, OR = 3.80, 95% CI (2.39, 6.08). 

Being on isoniazid preventive therapy (IPT) treatment was marginally associated with tuberculosis co-infection, OR = 1.40, 95% CI (0.93, 2.28), with *P* value = 0.09 (Tables 2 and 3). 

### 3.3. Environmental Related Characteristics

Eighty (67.2%) of the cases and 179 (75.2%) of the controls had less than five adults in the house hold. Higher proportion of the controls 213 (89.5%) had disposed waste outside of the compound when compared to the cases. Ninety-one (76.5%) of the cases and 143 (60.1%) of the controls house floor were made of soil. Having house made of soil floor was significantly associated with TB (*P* = 0.002) though did not reach statistically significant after controlling for confounders. Forty-seven (39.5%) of the cases and 60 (25.2%) of the controls did not have separated kitchen. The presence of separate kitchen decreased the chance of acquiring TB by 48%, OR = 0.52, 95% CI (0.32–0.82) (Table 4). 

### 3.4. Factors Independently Associated with TB/HIV Coinfection

Factors independently associated with TBHIV co-infection were having no formal education, divorced/widowed, underweight (BMI < 18.5 kg/m^2^), having history of diabetic mellitus, being in an advanced WHO clinical stage of HIV/AIDS. Having a separate kitchen was negatively associated with TB/HIV co-infection (Table 5).

## 4. Discussion

 In this study, being divorced or widowed, not attending formal education, being underweight, having history of diabetic mellitus, and being in advanced WHO clinical stage of HIV were factors associated with TB infection. Very importantly, having a separate kitchen from residential house had a protective effect from acquiring TB infection. 

We had obtained consistent finding with previous studies from Gambia and Guinea Bissau [10, 11] where patients who were divorced/widowed were at greater risk of TB/HIV co-infection. This can be seen in the view of marriage having a positive effect on health of an individual in a sense that those who get married and stayed together have advantages of better health as a result of positive psychological and social impacts [12]. Furthermore, evidence has shown that tuberculosis can lead to marriage disruption [13] so that they could be in divorced/widowed state during the study time which is difficult to ascertain by this study and left exposed for further research. 

Individuals who attended formal education are more likely efficient producers of health as they have higher awareness and can take precautionary measures to prevent health problems. We identified that those who have no formal education were more likely acquire TB infection than those who attended formal education. Studies from India, Gambia and Jimma (Ethiopia) also reported similar findings [6, 10, 14, 15].

Previous research indicated that low body mass index which explains undernutrition is significantly associated with TB infection [16, 17]. This might be due to the fact that undernutrition weakens the immunity level that increases the reactivation of latent tuberculosis infection. Building on the existing knowledge this study also found out that low body mass index increased the likelihood tuberculosis co-infection.

The other important finding identified was patients WHO clinical staging and TB-coinfection association. Those in third and fourth WHO clinical stages were about two times more likely to develop TB compared with those in WHO clinical stages one and two. Congruent findings have been reported before from in-country and outside of the country [6, 7, 18, 19]. This could be explained as once the patients get into late stages, the immunity protective capacity will be minimal which would make them prone to tuberculosis infection. A worth to mention as well is that TB is one of the ADIS defining criteria to categorize the patients in to the late WHO clinical staging which again was not captured with this study. 

Furthermore, having known history of diabetic mellitus was 3.6 times more likely associated with TB/HIV co-infection as compared to those who did not have known history of diabetic mellitus. Likewise, different studies reported similar findings [15, 20, 21]. This can be explained as diabetes mellitus-related complications increase the acquisition of TB and for people with both diseases the TB treatment is more ineffective [22].

Interestingly, this study also showed that having a separate kitchen decreased the risk of TB co infection by 52% compared with those who did not have. Similar findings had been reported from south India and in Jimma (Ethiopia) [6, 15]. This might be related with the indoor biomass use that could be associated with air pollution that could contribute to the respiratory infections including tuberculosis [23, 24].

Finally, this study has limitations. First, because of the nature of the study design, and data collection approaches, social desirablity bias is unavoidable. Secondly, recall bias is expected as some of the variables asked retrospectively. Lastly, reverse causal relationship might exist which could have an effect on the generalizability of the findings. 

## 5. Conclusions

The study came up with that educational and marital status, body mass index, WHO clinical staging, and having history of diabetic mellitus are associated with TB co-infection among adult HIV positives. Most of these factors can be intervened at individual and institutional levels though some like education and nutrition need a broader societal level integration to intervene. 

## Figures and Tables

**Table 1:** Sociodemographic factors associated with TB infection among HIV-positive adults in Nekemte town public health facilities, western Ethiopia, 2012.

Sociodemographic variables	Cases (*N* = 119)	Controls (*N* = 238)		*P* value
	*n* (%)	*n* (%)	COR	
Sex				
Male	58 (48.7)	107 (45)	1.16 (0.74, 1.80)	0.49
Female	61 (51.3)	131 (55)	1.00	
Age				
15–35 years	62 (52.1)	140 (58.8)	1.00	
>35 years	57 (47.9)	98 (41.2)	1.31 (0.84, 2.04)	0.22
Educational status				
No formal education	49 (41.2)	57 (23.9)	4.55 (2.09, 9.90)	0.000*
Primary education	40 (33.6)	63 (26.5)	3.34 (1.48, 5.24)	0.001*
Secondary education	20 (16.8)	65 (27.3)	1.35 (0.78, 2.34)	0.28
Tertiary education	10 (8.4)	53 (22.3)	1.00	
Marital status				
Single	21 (17.6)	73 (30.7)	1.00	
Married	54 (45.4)	109 (45.8)	1.70 (0.95, 2.64)	0.78
Divorced/widowed	44 (37)	56 (23.5)	2.73 (1.49, 5.34)	0.002*
Employment status				
Employed	42 (35.3)	113 (43.4)	1.00	
Unemployed	77 (64.7)	125 (52.5)	1.65 (1.05, 2.61)	0.02*
Monthly income				
<650 ETB**	61 (51.3)	116 (48.7)	1.10 (0.71, 1.71)	0.65
≥650 ETB	58 (48.7)	122 (51.3)	1.00	
Residence				
Urban	111 (93.3)	218 (91.6)	1.27 (0.54, 2.98)	0.57
Rural	8 (6.7)	20 (8.4)	1.00	

*Statistically significant.

**1 USD = 18.5 ETB in 2012.

**Table 2:** Clinical variable associated with TB infection among HIV-positive adults in Nekemte town public health facilities, western Ethiopia, 2012.

Clinical related variable	Cases (*N* = 119)	Controls (*N* = 238)		*P* value
	*n* (%)	*n* (%)	COR	
WHO clinical stage				
Stages I and II	43 (36.1)	129 (54.2)	1.00	
Stages III and IV	76 (63.9)	109 (45.8)	2.09 (1.33, 3.28)	0.001*
Haemoglobin level				
<10	13 (10.9)	13 (5.5)	2.96 (1.28, 6.80)	0.01*
10–12.49	56 (47.1)	77 (32.4)	2.08 (0.59, 3.19)	0.45
≥12.5	50 (42)	148 (62.2)	1.00	
CD4 count				
<200	33 (27.7)	41 (17.2)	2.35 (1.23, 4.48)	0.009*
200–499	61 (51.3)	124 (52.1)	1.48 (0.94, 2.84)	0.08
≥500	25 (21)	73 (30.7)	1.00	
BMI				
<18.5	67 (56.3)	60 (25.2)	3.80 (2.39, 6.08)	<0.001*
≥18.5	52 (43.7)	178 (74.8)	1.00	

*Statistically significant.

**Table 3:** Host factors associated with TB infection among HIV-positive adults in Nekemte town public health facilities, western Ethiopia, 2012.

Host variables	Cases (*N* = 119)	Controls (*N* = 238)		*P* value
	*n* (%)	*n* (%)	COR	
Smoking				
Never	93 (78.21)	92 (80.7)	1.00	
Past	19 (16)	29 (12.2)	1.35 (0.69, 2.65)	0.34
Current	7 (5.9)	17 (7.1)	0.85 (0.31, 2.27)	0.72
Asthma				
Yes	13 (10.9)	21 (8.8)	1.26 (0.61, 2.62)	0.52
No	106 (89.1)	217 (91.2)	1.00	
Diabetic mellitus				
Yes	16 (13.4)	10 (4.2)	3.54 (1.55, 8.07)	0.002*
No	103 (86.6)	228 (95.8)	1.00	
Taking ART				
Yes	77 (64.7)	146 (61.3)	1.15 (0.73, 1.82)	0.53
No	42 (35.3)	92 (38.7)	1.00	
Taking IPT				
Yes	46 (38.7)	114 (59.2)	1.00	
No	73 (61.3)	124 (40.8)	1.40 (0.93, 2.28)	0.09
Previous history of TB				
Yes	32 (26.9)	46 (19.3)	1.53 (0.91, 2.57)	0.10
No	87 (73.1)	192 (80.7)	1.00	
Presence of TB (family)				
Yes	36 (30.3)	57 (23.9)	1.37 (0.84, 2.25)	0.20
No	83 (69.7)	181 (76.1)	1.00	
History of pneumonia				
Yes	17 (14.3)	14 (5.9)	2.60 (1.26, 5.61)	0.01*
No	102 (85.7)	224 (94.1)	1.00	
History of RTI				
Yes	20 (16.8)	38 (16)	1.06 (0.58, 1.92)	0.83
No	99 (83.2)	200 (84)	1.00	

*Statistically significant.

**Table 4:** Environmental factors associated with TB infection among HIV-positive adults in Nekemte town public health facilities, western Ethiopia, 2012.

Environmental variables	Cases (*N* = 119)	Controls (*N* = 238)		*P* value
	*n* (%)	*n* (%)	COR	
Wall of house				
Mud/mud brick	102 (85.7%)	191 (80.3)	1.47 (0.80, 2.70)	0.26
Cement	17 (14.3%)	47 (19.7)	1.00	
Separate kitchen				
Yes	41 (34.5%)	117 (49.2)	0.549 (0.34, 0.85)	0.009*
No	78 (65.5%)	121 (50.8)	1.00	
Waste disposal site				
In the compound	18 (15.1%)	25 (10.5)	1.51 (0.79, 2.91)	0.20
Outside	101 (84.5%)	213 (89.5)	1.00	
Floor of house				
Earth	91 (76.5)	143 (60.1)	2.15 (1.31, 3.54)	0.002*
Cement	28 (23.5)	95 (39.9)	1.00	
PPR				
<1	15 (12.6)	37 (15.5)	1.00	
1-2	87 (73.1)	167 (70.2)	1.29 (0.64, 2.61)	0.45
>2	17 (14.3)	34 (14.3)	1.23 (0.78, 1.93)	0.62
Ceiling				
Yes	74 (62.2)	136 (57.1)	1.23 (0.60, 2.25)	0.55
No	45 (37.8)	102 (42.9)	1.00	
Number of windows				
0	13 (10.9)	16 (6.7)	1.47 (0.63, 3.41)	0.32
1	15 (12.6)	57 (23.9)	0.48 (0.24, 0.92)	0.20
>2	91 (76.5)	165 (69.3)	1.00	

*Statistically significant.

**Table 5:** Factors independently associated with TB infection among HIV-positive adults in Nekemte town public health facilities, western Ethiopia, 2012.

Independent predictor	COR	AOR	*P* value
Marital status			
Single	1.00	1.00	
Married	1.70 (0.95, 2.64)	1.82 (0.66, 2.93)	0.49
Divorced/widowed	2.73 (1.49, 5.34)	3.02 (1.70, 7.88)	0.001*
Educational status			
No formal education	4.55 (2.09, 9.90)	4.32 (2.20, 14.15)	<0.001*
Primary education	3.34 (1.48, 5.24)	2.90 (0.88, 4.09)	0.09
Secondary education	1.36 (0.78, 2.34)	1.24 (0.62, 2.46)	0.53
Tertiary education	1.00	1.00	
Diabetic mellitus			
Yes	3.54 (1.55, 8.07)	3.63 (1.33, 9.94)	0.01*
No	1.00	1.00	
BMI (kg/m^2^)			
<18.5	3.80 (2.39, 6.08)	3.87 (2.18, 6.87)	0.01*
≥18.5	1.00	1.00	
WHO clinical stage			
Stages I and II	1.00	1.00	
Stages III and IV	2.09 (1.33, 3.28)	2.29 (1.32, 3.98)	0.003*
Separate kitchen			
Yes	0.52 (0.32, 0.82)	0.48 (0.28, 0.81)	0.007*
No	1.00	1.00	

*Statistically significant.
